# Polymorphism of Pb_5_(PO_4_)_3_OH_δ_ within the LK-99 mixture

**DOI:** 10.1107/S2052520624010023

**Published:** 2024-11-19

**Authors:** Mingyu Xu, Haozhe Wang, Cameron Vojvodin, Jayasubba Reddy Yarava, Tuo Wang, Weiwei Xie

**Affiliations:** a Department of Chemistry Michigan State University East Lansing MI48824 USA; IISER Kolkata, India

**Keywords:** orthorhombic apatite, superconductivity, LK-99

## Abstract

During the synthetic exploration targeting the polycrystalline compound LK-99, an unexpected phase, Pb_5_(PO_4_)_3_OH_δ_, was identified as a byproduct. We elucidated the composition of this compound through single-crystal X-ray diffraction analysis. Subsequent synthesis of the target compounds was achieved via high-temperature solid-state pellet reactions. The newly identified Pb_5_(PO_4_)_3_OH_δ_ has an orthorhombic crystal structure with space group *Pnma*, representing a unique structure differing from the hexagonal apatite phases of Pb_10_(PO_4_)_6_O and Pb_5_(PO_4_)_3_OH. Comprehensive temperature- and magnetic-field-dependent magnetization studies unveiled a temperature-independent magnetic characteristic of Pb_5_(PO_4_)_3_OH_δ_. Solid-state nuclear magnetic resonance spectroscopy was employed to decipher the origins of the phase stability and confirm the presence of hydrogen atoms in Pb_5_(PO_4_)_3_OH_δ_. These investigations revealed the presence of protonated oxygen sites, in addition to the interstitial water molecules within the structure, which may play critical roles in stabilizing the orthorhombic phase.

## Abstract 2

A new orthorhombic crystal Pb_5_(PO_4_)_3_OH_δ_ of space-group symmetry *Pnma* significantly differs differing from the hexagonal apatite phases of Pb_10_(PO_4_)_6_O and Pb_5_(PO_4_)_3_OH.

## 1. Introduction

After the claim of the discovery of an ambient-pressure room-temperature superconductor (*T*_c_ > 400 K) LK-99, (Pb_10−*x*_Cu*_x_*)(PO_4_)_6_O (Lee, Kim & Kwon, 2023 ▸), with a subsequent report of a levitation experiment at room temperature indicating strong diamagnetic signals (Lee, Kim, Kim *et al.*, 2023 ▸), LK-99 has garnered unprecedented attention. Despite numerous attempts by various research groups to replicate and verify the superconductivity of LK-99 (Zhu *et al.*, 2023 ▸; Timokhin *et al.*, 2023 ▸; Kumar *et al.*, 2023 ▸; Wu *et al.*, 2023 ▸; Hou *et al.*, 2023 ▸), increasing experimental evidence has begun to cast doubt on its superconducting nature (Garisto, 2023 ▸).

In the quest to unravel the mysteries of LK-99’s superconductivity, our investigation has led to the discovery of several new phases. Among these, the novel structure of hy­droxy­lpyromorphite, Pb_5_(PO_4_)_3_OH_δ_, stands out and is different from the conventional hexagonal phase known for Pb_10_(PO_4_)_6_O (Brückner *et al.*, 1995 ▸; Kim *et al.*, 1997 ▸; Barinova *et al.*, 1998 ▸). The compound Pb_10_(PO_4_)_6_O has been termed oxypyromorphite, a nomenclature that underscores its structural resemblance to pyromorphite, Pb_5_(PO_4_)_3_Cl, and hy­droxy­lpyromorphite, Pb_5_(PO_4_)_3_(OH), which adopts an apatite-like hexagonal structure, wherein O^2−^ anions substitute for the halide ions typically found in apatite structures. This substitution suggests the presence of vacancies at some halide sites, in contrast to the original proposition of a (Pb^2+^)_9_(Pb^4+^)(PO_4_)_6_O_2_ formula (Ito, 1968 ▸), which would negate the need for such vacancies. However, further studies confirmed the absence of Pb^4+^ cations in oxypyromorphite (Merker *et al.*, 1970 ▸). Despite its intriguing properties, detailed crystal structure analysis of oxypyromorphite has yet to be carried out. In addition, the lead-based compounds Pb_4_O(PO_4_)_2_, Pb_8_O_5_(PO_4_)_2_ and Pb_10_(PO_4_)_6_O have been studied for several decades (Yang *et al.*, 2001 ▸; Brixner & Foris, 1973 ▸; Krivovichev & Burns, 2003 ▸). The ferro-elastic Pb_8_O_5_(PO_4_)_2_ and its vanadium analog Pb_8_O_5_(VO_4_)_2_ (Dudnik & Kolesov, 1980 ▸; Kiosse *et al.*, 1982 ▸) have also been discovered. Although X-ray and optical analyses on the single crystals of these compounds have been conducted, the crystal structures of both remain unresolved.

In this study, we present a synthetic strategy for the new Pb_5_(PO_4_)_3_OH_δ_ compound using a high-temperature solid-state pellet reaction. Millimetre-sized single crystals were obtained from the reaction. Single-crystal and powder X-ray diffraction (XRD) experiments were conducted to determine the crystal structure and confirm the phase information. Accordingly, Pb_5_(PO_4_)_3_OH_δ_ was found to crystallize in the orthorhombic crystal system with the space group *Pnma*. Different from the hexagonal apatite phase Pb_10_(PO_4_)_6_O with the balanced charge of (Pb^2+^)_10_(PO_4_^3−^)_6_O^2−^, the compound of Pb_5_(PO_4_)_3_O cannot be charge-balanced with the sole existence of Pb^2+^ and full occupancies on all atomic sites. To confirm the existence of a proton (H^+^) to balance the charge in Pb_5_(PO_4_)_3_OH_δ_, high-magnetic-field (800 MHz or 18.8 T) solid-state nuclear magnetic resonance (NMR) spectroscopy was used to determine the chemical environments of proton sites and the presence of water molecules in the system.

## 2. Synthesis and experimental methods

### 2.1. Chemical synthesis

Pb_5_(PO_4_)_3_OH_δ_ crystals were synthesized in two steps. The first step was synthesis of the precursors. The mixture of PbO (99.3%, BAKER ANALYZED) and PbSO_4_ (99.1%, BAKER ANALYZED) was heated under a vacuum for 24 h with 1:1 mole ratio. After the reaction, we obtained the Pb_2_(SO_4_)O precursor with Pb_3_(SO_4_)O_2_ (∼1.5 at.%]) impurity. Another precursor is Cu_3_P, obtained by heating Cu powder (99.9%, Alfa Aesar) and P powder (99%, Beantown Chemical) for 48 h at 550°C under vacuum. The molar ratio of Cu and P was 3:1 and the powder XRD results of the two precursors are shown in the supporting information (Figs. S1a and S1b). The second step was mixing Pb_2_(SO_4_)O and Cu_3_P in the ratio 5:3, remaining at 925°C for 20 h. Millimetre-size single crystals of Pb_5_(PO_4_)_3_OH_δ_ were obtained. All the reaction products were powder pressed using a 0.25 inch (internal diameter) dry pellet pressing die made of carbon steel. A 2 ml alumina cylindrical crucible held the pressed pellet before it was sealed in a fused silica tube under vacuum with around 30 mTorr pressure. After the solid-state reaction, transparent single-crystalline samples can easily be identified on the bottom pellet, which can be separated from black-colored polycrystalline chunks and copper-colored solidified drops on the surface of the black chunks. The rest of the measurements were done using the single crystals separated mechanically from the mixture.

### 2.2. Structure determination and phase analysis

Pb_5_(PO_4_)_3_OH_δ_ forms transparent, rod-like, brittle single crystals. A single crystal was selected, mounted on a nylon loop with Paratone oil and measured using an XtalLAB Synergy, Dualflex, Hypix single-crystal X-ray diffractometer. Data were collected using ω scans with Mo *K*α radiation (λ = 0.71073 Å) and Ag *K*α radiation (λ = 0.56087 Å), a micro-focus sealed X-ray tube, 65 kV, 0.67 mA. The total number of runs and images was based on the strategy calculation from the *CrysAlisPro* (Rigaku OD, 2017 ▸) program. Data reduction was performed with correction for Lorentz polarization. A numerical absorption correction was applied based on Gaussian integration over a multifaceted crystal model (Parkin *et al.*, 1995 ▸). Empirical absorption correction used spherical harmonics, implemented in the *SCALE3 ABSPACK* scaling algorithm (Walker & Stuart, 1983 ▸). The structure was solved and refined using the *SHELXTL* software package (Sheldrick, 2015*a* ▸, Sheldrick, 2015*b* ▸). Tables 1 ▸ and 2 ▸ show the results of the single-crystal XRD. For the powder XRD measurements, single crystals were ground in an agate mortar and pestle, and the powder placed onto the 20 × 20 × 0.5 mm Rigaku Square groove. Powder XRD measurements were carried out using a Rigaku MiniFlex powder diffractometer in Bragg–Brentano geometry with Cu *K*α radiation (λ = 1.5406 Å). Room-temperature measurements were performed with a step size of 0.01° at a scan speed of 0.5° per minute over a Bragg angle (2θ) range of 10–90°. *GSAS II* (Toby & Von Dreele, 2013 ▸) was used to perform the Rietveld refinement and analyze phase information.

### 2.3. Solid-state NMR spectroscopy for detecting protons

^1^H solid-state NMR experiments were conducted on a Bruker NEO spectrometer with a narrow-bore magnet with *B*_0_ = 18.8 T [ν_0_(^1^H) = 800 MHz] at room temperature (298 K). Spectra were acquired using a Phoenix NMR 1.6 mm HXY magic-angle spinning (MAS) probe with samples packed into 1.6 mm (outer diameter) zirconia rotors. The MAS frequency was set to 8 kHz. ^1^H chemical shifts were referenced to alanine (δ_iso_ = 1.38 p.p.m.) as a secondary reference with respect to tetra­methyl­silane (δ_iso_ = 0 p.p.m.). All ^1^H spectra were acquired using a rotor-synchronized Hahn echo (90°–τ–180° acquisition) with 2.5 µs (100 kHz) π/2 pulses, an interpulse delay (τ) of 500 µs and a recycle delay of 2 s (see Table S1 for further details). Spectra were processed in *TopSpin 4.1* (Bruker), and simulations of all spectra were prepared using *ssNake* v1.3 (van Meerten *et al.*, 2019 ▸).

### 2.4. Magnetic measurements

Temperature- and magnetic-field-dependent DC and vibrating sample magnetometry (VSM) magnetization data were collected using a Quantum Design Magnetic Property Measurement System (MPMS3). Temperature- and magnetic-field-dependent DC magnetization measurements were taken on the powder sample loaded in the powder sample holder and put into a brass half-tube sample holder. In VSM measurements, a peak amplitude of 5 mm and an average of 2 s were used. An empty powder sample holder was measured under the same conditions to estimate the background to be subtracted from the measurements.

## 3. Results and discussion

**Figure 1:**
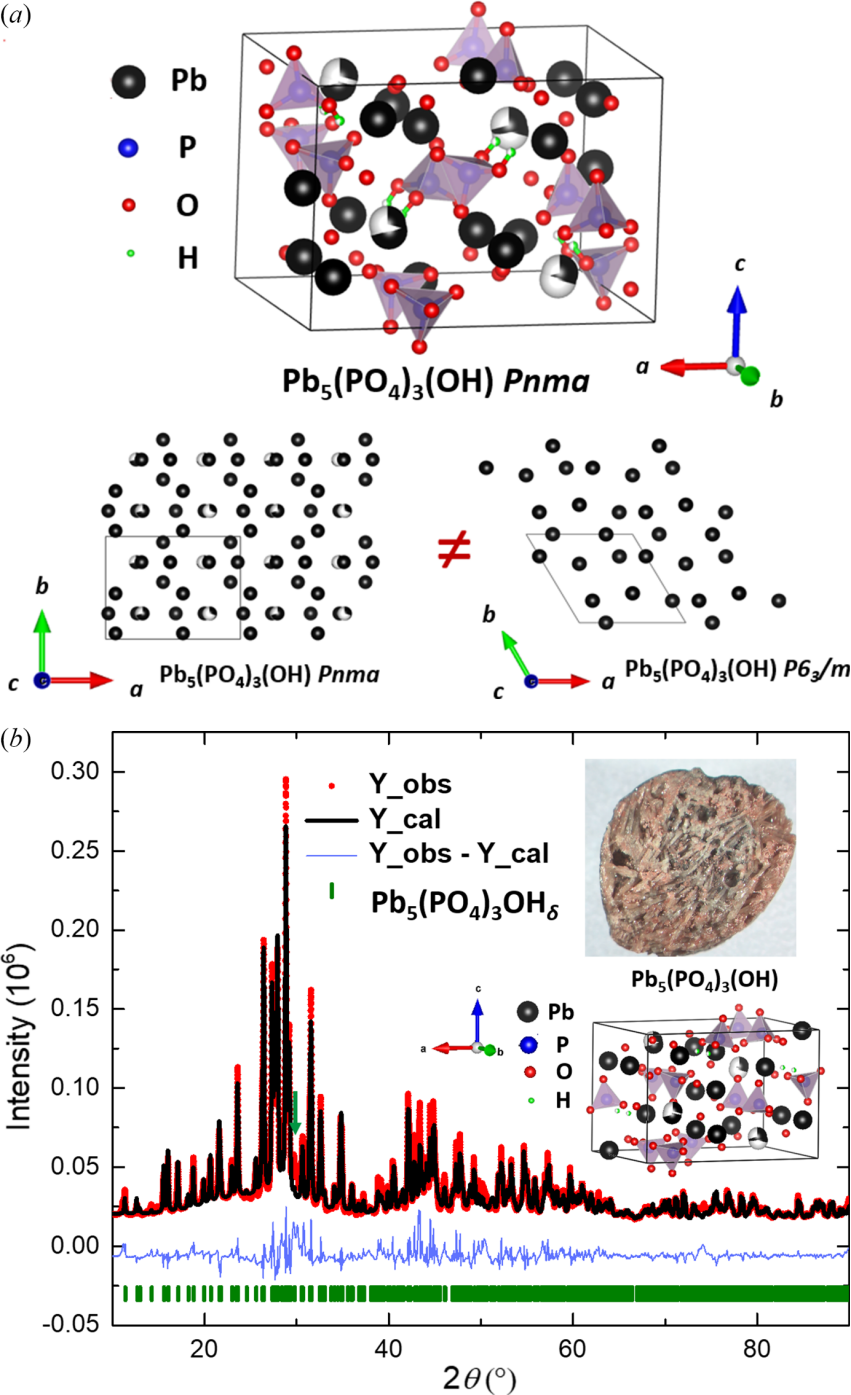
The crystal structure and the powder XRD data of Pb_5_(PO_4_)_3_OH_δ_. (*a*) shows the crystal structure of Pb_5_(PO_4_)_3_(OH) from single-crystal XRD and a comparison of Pb atom distribution between the orthorhombic and hexagonal structures. (*b*) Powder XRD data of Pb_5_(PO_4_)_3_OH_δ_ from in-laboratory diffraction measurements. The experimental data are plotted as red dots. The black line gives the Rietveld refinement. The blue line indicates the corresponding residual pattern (difference between observed and calculated patterns). Bars give Pb_5_(PO_4_)_3_OH_δ_ peak positions from single-crystal XRD measurement. The green arrow indicates the peak from an impurity. On the upper right of the figure, a picture of the crystals is shown; the size of the chunk is about 5 mm.

The crystal structure of Pb_5_(PO_4_)_3_OH_δ_ exhibits similarities to apatite-like lead compounds, including pyromorphite [Pb_5_(PO_4_)_3_Cl], hy­droxy­lapatite [Pb_5_(PO_4_)_3_(OH)] and the LK-99 precursor, Pb_10_(PO_4_)_6_O. Previous determinations of hy­droxy­lapatite’s structure, through neutron and X-ray powder diffraction analysis, identified the OH group positioned at the 4*e* Wyckoff site with a 0.5 site occupation factor (Kim *et al.*, 1997 ▸). In contrast, Barinova *et al.*’s refinement using single-crystal diffraction data located the OH group at the 2*b* site, indicating full occupancy – a characteristic more aligned with the halide ion positions in pyromorphite-like compounds (Pb_5_(PO_4_)_3_*X*, where *X* = F, Cl (Barinova *et al.*, 1998 ▸). The structural framework of Pb_10_(PO_4_)_6_O mirrors that of hy­droxy­lapatite, with the O4 atom situated at the 4*e* site, albeit with a reduced occupation factor of 0.25. Our investigation into a single crystal of Pb_5_(PO_4_)_3_OH_δ_ revealed a deviation from the expected hexagonal structure to an orthorhombic *Pnma* space group, attributed to lattice parameter distortions, as shown in Fig. 1 ▸(*a*). The hydrogen atoms were refined with half occupancy at the 8*d* sites. However, considering the limitations of XRD in accurately detecting hydrogen positions, we employed high-field ^1^H solid-state NMR (ssNMR) to further elucidate the hydrogen occupancies, providing a more comprehensive understanding of the structural intricacies of Pb_5_(PO_4_)_3_OH_δ_. To ascertain the phase purity and facilitate the investigation of its physical properties, powder XRD analysis was performed, with the results presented in Fig. 1 ▸(*b*). The data were refined using Rietveld refinement, and the green peak suggests a peak from a minor impurity phase.

**Figure 2:**
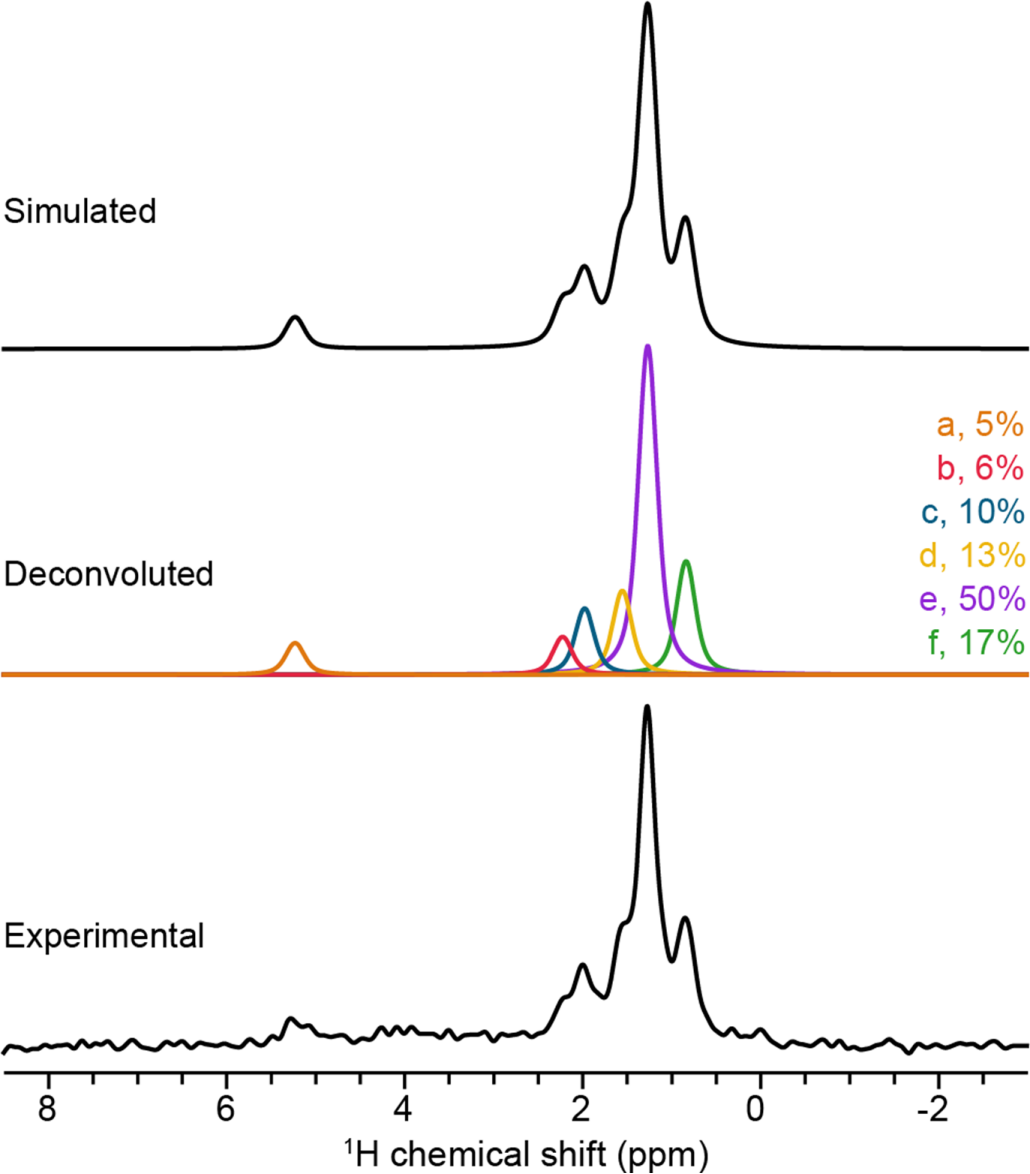
^1^H MAS solid-state NMR analysis of Pb_5_(PO_4_)_3_OH_δ_. Experimental ^1^H MAS NMR of Pb_5_(PO_4_)_3_OH_δ_ is shown in blue, with corresponding analytical simulations in black, and deconvolution of the simulation is shown in color. Six ^1^H resonances are color-coded and labeled as *a*–*f*, with their relative intensity percentages provided.

The ^1^H ssNMR spectrum features six underlying peaks with their isotropic chemical shifts (δ_iso_) ranging from 0.8 to 5.2 p.p.m. in a 1:1.2:2.1:2.7:10.4:3.6 ratio from left to right (Fig. 2 ▸ and Table 3 ▸). This is surprising since the solved single-crystal XRD structure only includes one hydrogen position, while the NMR data indicate a far more complex ^1^H environment. It is not unusual for ssNMR to detect additional structural features that are invisible to XRD techniques (Morris *et al.*, 2017 ▸; Zhang *et al.*, 2022 ▸; Inukai *et al.*, 2016 ▸; Corlett *et al.*, 2019 ▸; Li *et al.*, 2013 ▸; Serrano-Sevillano *et al.*, 2019 ▸). A few possible explanations for these hydrogen resonances are: (i) structural defects in the sample where the phosphate ion reacts with atmospheric water, (ii) water being incorporated into the structure, either occupying vacancies in the octahedral or tetrahedral holes in the crystal structure and/or (iii) atmospheric water being bound to the lead as a ligand.

Since only a single spectrum for Pb_5_(PO_4_)_3_OH_δ_ was acquired with no internal reference, only approximate estimation of the amount of hydrogen in the sample is possible. Precise quantification would require a low radiofrequency pulse, a different pulse sequence (*i.e.* Bloch decay), accurate site assignments of all hydrogen atoms and a series of standards to construct a calibration curve (Bharti & Roy, 2012 ▸; Pauli *et al.*, 2012 ▸; Giraudeau, 2017 ▸; Holzgrabe, 2010 ▸; Pauli *et al.*, 2015 ▸). Hence, we are limited to approximately quantifying the Pb_5_(PO_4_)_3_OH_δ_ spectrum relative to another ^1^H NMR spectrum of a known sample. First, a ^1^H spectrum of alanine with the exact same experimental parameters as Pb_5_(PO_4_)_3_OH_δ_ was acquired using a sufficiently long recycle delay (in this case, 2 s) to completely re-equilibrate the magnetization before the next scan. Second, a Hahn echo with a long interpulse delay (in this case, 500 µs) was used for these experiments in order to eliminate the broad ^1^H background signal from the probe to allow for easier quantification of the spectra. It has been shown that the relative quantification of NMR spectra of two compounds is possible using the following equation (Malz & Jancke, 2005 ▸):
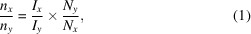
where *n_x_*/*n_y_* is the molar ratio, *I_x_*/*I_y_* is the total integrated intensity ratio and *N* is the number of nuclei corresponding to the resonance line. Using the total integrated intensities from a spectrum of alanine and Pb_5_(PO_4_)_3_OH_δ_, we are able to write equation (2) for approximately quantifying the spectrum as
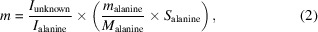
where *I* is the total integrated intensity of the spectrum, *M* is the molecular weight, *m* is the mass of the sample packed in the NMR rotor and *S* is the number of moles of hydrogen in the sample. Using equation (2), we were able to estimate the amount of hydrogen in Pb_5_(PO_4_)_3_OH_δ_ to be approximately 82.5 µg or 1.15 wt%.

**Figure 3:**
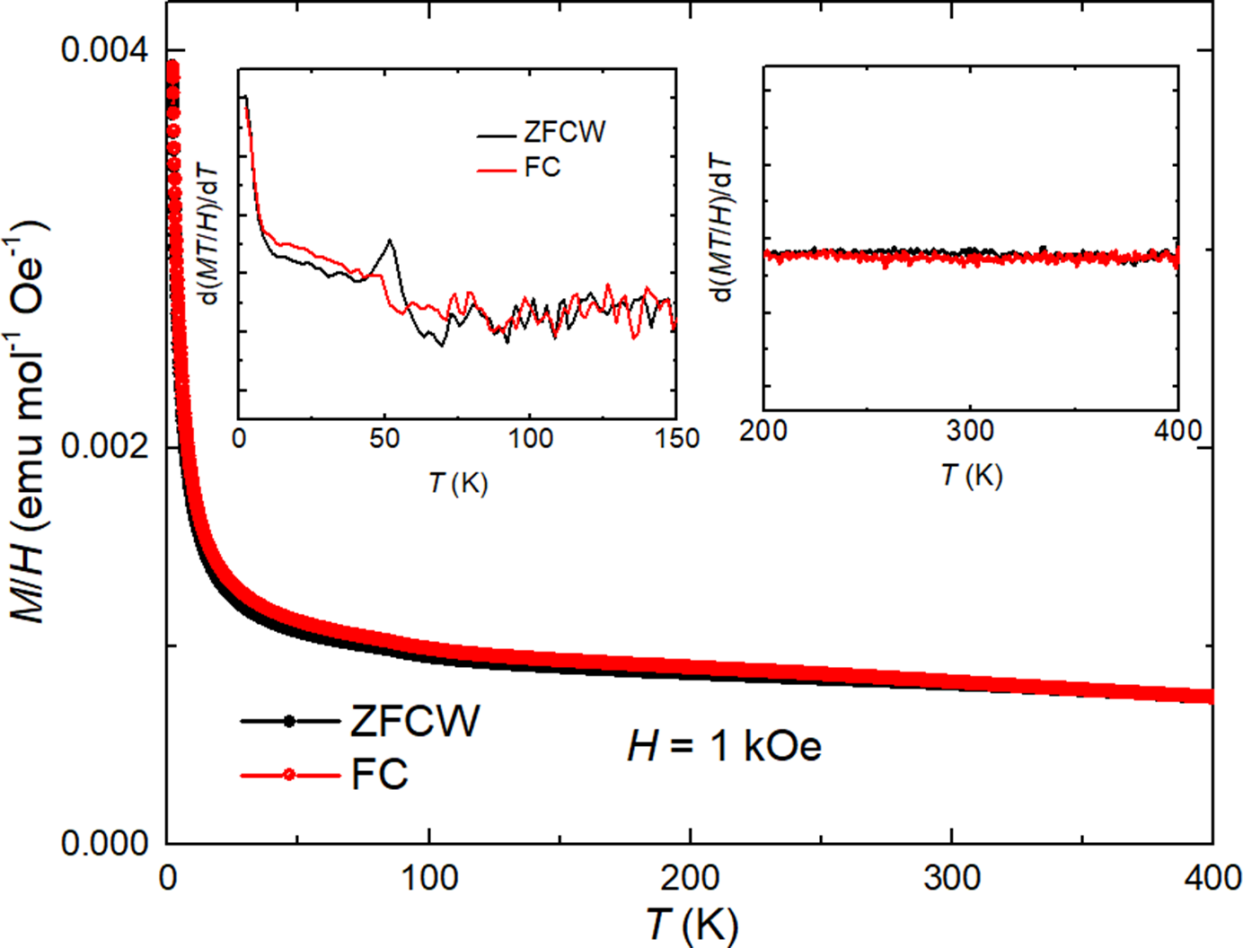
Temperature-dependent magnetization of Pb_5_(PO_4_)_3_OH_δ_. ZFCW and FC magnetization as a function of temperature is given by VSM in the range of 1.8–400 K with a field of 1 kOe. The left inset shows d(*MT*/*H*)/d*T* as a function of temperature in the low-temperature range. The right inset shows the derivative of d(*MT*/*H*)/d*T* in the high-temperature range.

While Fig. 1 ▸(*b*) depicted the primary phase of the transparent single crystal, Pb_5_(PO_4_)_3_OH_δ_, the data also suggested the presence of minor impurity phases within the sample. Single-crystal and powder XRD analyses have identified Cu_2_S as the predominant impurity. Prior to evaluating the magnetization data, it is essential to consider the potential magnetic contributions from Cu_2_S impurities. If Cu_2_S were to significantly influence the magnetic behavior, one might expect to observe a β-to-γ phase transition around 370 K in resistivity, heat capacity and magnetic susceptibility measurements (Zhu *et al.*, 2023 ▸). VSM measurements, conducted without background subtraction across a temperature range of 1.8 to 400 K and presented in Fig. 3 ▸, do not reveal any magnetic transition near 370 K. The large positive magnetization is also different from previous work (Zhu *et al.*, 2023 ▸). The presence of undetected impurity phases, a common occurrence in the solid-state synthesis of LK-99, cannot be overlooked. Fig. 3 ▸ presents the findings from temperature-dependent magnetization investigations, conducted under identical experimental conditions to those applied to an empty sample holder to guarantee precision. The magnetization profiles depicted in Fig. 3 ▸, obtained through zero-field-cooled-warming (ZFCW) and field-cooled (FC) methods, exhibit distinctive Curie–Weiss-like behavior fitting is shown in Figs. S2a and S2b). A significant deviation between the ZFCW and FC data is evident around 50 K, where a kink-like anomaly, potentially arising from oxygen trapped in the measured sample, is identified in the ZFCW magnetization trajectory. This anomaly is accentuated in the derivative d(*MT*/*H*)/d*T* plot, prominently featured in the inset at the upper-left corner of the figure, further emphasizing its significance. The field-dependent magnetization is also shown in Fig. S3b.

In conclusion, the orthorhombic phase of Pb_5_(PO_4_)_3_OH_δ_ was successfully synthesized via solid-state reaction techniques with the objective of creating LK-99. The determination of the phase composition and crystal structure of the product was achieved through an integrated approach, utilizing both single-crystal and powder X-ray diffraction analyses, complemented by solid-state NMR spectroscopy.

## Supplementary Material

Crystal structure: contains datablock(s) I. DOI: 10.1107/S2052520624010023/rm5078sup1.cif

Structure factors: contains datablock(s) I. DOI: 10.1107/S2052520624010023/rm5078Isup2.hkl

Rietveld powder data: contains datablock(s) Paper_LK99_2_20230905_2.bak166. DOI: 10.1107/S2052520624010023/rm5078Isup3.rtv

Supplementary figures and table. DOI: 10.1107/S2052520624010023/rm5078sup4.pdf

CCDC reference: 2391128

## Figures and Tables

**Table 1:** Experimental details Values in parentheses are the estimated standard deviation from refinement.

Chemical formula	Pb_5_(PO_4_)_3_OH
Formula weight (g mol^−1^)	1337.87
Space group	*Pnma*
Unit-cell dimensions (Å)	*a* = 13.5137 (4), *b* = 10.2904 (4), *c* = 9.3838 (3)
Volume (Å^3^)	1304.93 (7)
*Z*, *Z*′	4, 0.5
Density (calculated) (g cm^−3^)	6.810
Absorption coefficient μ (mm^−1^)	64.725
Crystal size (mm)	0.076 × 0.056 × 0.049
*F*(000)	2240
	
Data collection	
2θ range (°)	5.286–82.472
No. of reflections collected	48595
No. of independent reflections	4458
*R*_int_	0.1257
	
Refinement method	Full-matrix least-squares on *F*^2^
No. of data, restraints, parameters	4458, 0, 114
Final *R* indices	*R*_1_ [*I* > 2σ(*I*)] = 0.0402; *wR*_2_ [*I* > 2σ(*I*)] = 0.0681 *R*_1_ (all) = 0.0811; *wR*_2_ (all) = 0.0758
Δρ_max_, Δρ_min_ (e Å^−3^)	+3.79 and −5.99
RMS deviation from mean (e Å^−3^)	0.511
Goodness-of-fit on *F*^2^	1.047

**Table 2:** Atomic coordinates and equivalent isotropic atomic displacement parameters (Å^2^) of Pb_5_(PO_4_)_3_OH *U*_eq_ is one-third of the trace of the orthogonalized *U_ij_* tensor. Values in parentheses are the estimated standard deviation from refinement.

Atoms	Wyckoff site	*x*	*y*	*z*	Occ.	*U*_eq_
Pb1	8*d*	0.07377 (2)	0.55577 (2)	0.29630 (2)	1	0.019 (1)
Pb2	4*c*	0.02043 (2)	0.75	−0.02843 (3)	1	0.017 (1)
Pb3	4*c*	0.23350 (3)	0.75	−0.30356 (4)	1	0.025 (1)
Pb4	4*c*	0.2750 (2)	0.75	0.1556 (2)	0.71	0.0285 (2)
Pb5	4*c*	0.2703 (5)	0.75	0.1277 (6)	0.29	0.05 (1)
P1	8*d*	−0.1631 (1)	0.5324 (1)	0.07074 (1)	1	0.0123 (2)
P2	4*c*	0.0030 (2)	0.75	−0.4252 (2)	1	0.0153 (4)
O1	4*c*	−0.0913 (6)	0.75	−0.34610 (8)	1	0.0402 (2)
O2	4*c*	−0.071 (5)	0.75	0.4179 (7)	1	0.0260 (1)
O3	4*c*	0.1089 (4)	0.75	0.1753 (5)	1	0.013 (1)
O4	8*d*	−0.1751 (4)	0.3859 (4)	0.0955 (4)	1	0.0248 (9)
O5	8*d*	−0.1685 (4)	0.5588 (5)	−0.0902 (5)	1	0.030 (1)
O6	8*d*	−0.0618 (4)	0.5794 (5)	0.1249 (6)	1	0.031 (1)
O7	8*d*	−0.2451 (4)	0.6053 (5)	0.1488 (6)	1	0.033 (1)
O8	8*d*	0.0635 (4)	0.8677 (5)	−0.3915 (7)	1	0.041 (1)
H	8*d*	0.0993	0.85244	−0.322954	0.5	0.061

**Table 3:** Isotropic chemical shifts (δ_iso_) and percentages of ^1^H resonances from ssNMR

	Peak					
Parameter	*a*	*b*	*c*	*d*	*e*	*f*
δ_iso_ (p.p.m.)	5.2	2.2	2.0	1.5	1.3	0.8
Percentage (%)	5	6	10	13	50	17
