# Supplementary material for: Polymorphism of Pb5(PO4)3OHδ within the LK-99 mixture
Source: Acta Crystallogr B Struct Sci Cryst Eng Mater. 2024 Nov 19;80(Pt 6):746–50. doi: 10.1107/S2052520624010023 (PMC11789162; doi:10.1107/S2052520624010023)
Supplement: Supplementary file 4 [file b-80-00746-sup4.pdf]

STRUCTURAL SCIENCE
CRYSTAL ENGINEERING
MATERIALS

Volume 80 (2024)

Supporting information for article:

Polymorphism of Pb5(PO4)3OHδ within the LK-99 mixture

Mingyu Xu, Haozhe Wang, Cameron Vojvodin, Jayasubba Reddy Yarava, Tuo Wang and WeiWei Xie

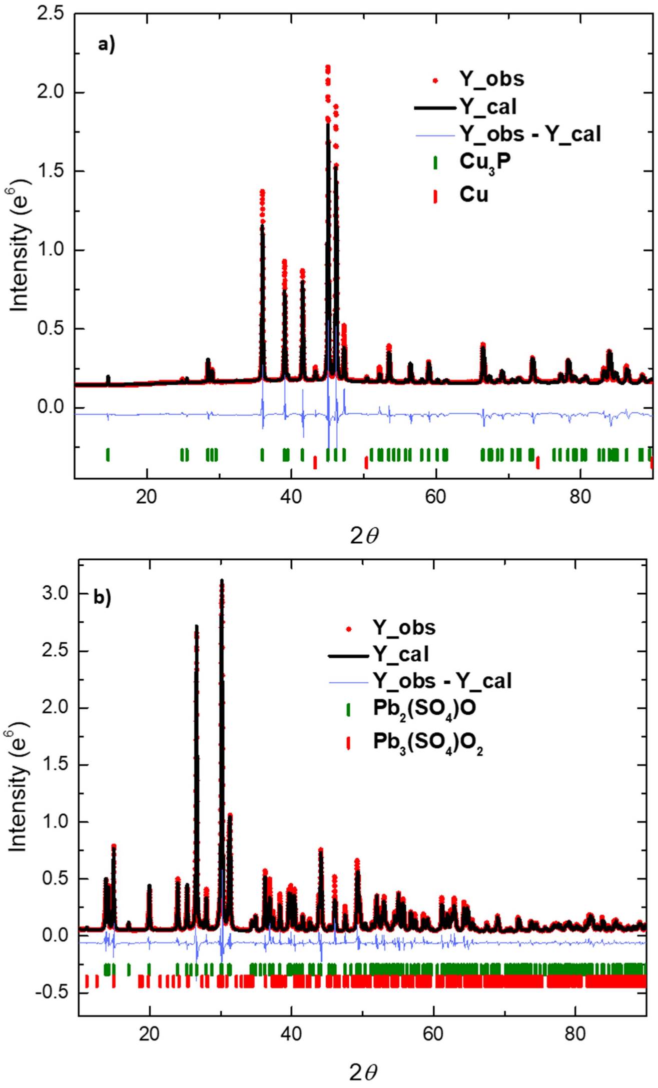

Figure S1 Power X-ray diffraction data of Cu_3P and $Pb_2(SO_4)O$. Powder X-ray diffraction data of **a**) Cu_3P and **b**) $Pb_2(SO_4)O$ precursor from in-lab diffraction measurements. The experimental data is plotted as red dots. The black line gives the Rietveld refinement. The blue line indicates the corresponding residual pattern (difference between observed and calculated patterns). Bars give the peak positions of different phases. Figure S1**a** shows the powder X-ray diffraction measurement of the pre-reacted Cu_3P precursor. Powder X-ray diffraction measurement is carried out to confirm the composition of the synthesized Cu_3P. Cu_3P, as well as a very small amount of Cu associated with non-fully reacted Cu, have been identified. The excess of copper is due to the loss of phosphorus during heating. About 6% (mole ratio) of Cu exists in the precursor. Powder X-ray diffraction measurement of pre-reacted $Pb_2(SO_4)O$ precursor is shown in Fig. 1b. According to the Rietveld refinement, $Pb_2(SO_4)O$ is the majority phase with about 1.5% (mole ratio) of $Pb_3(SO_4)O_2$.

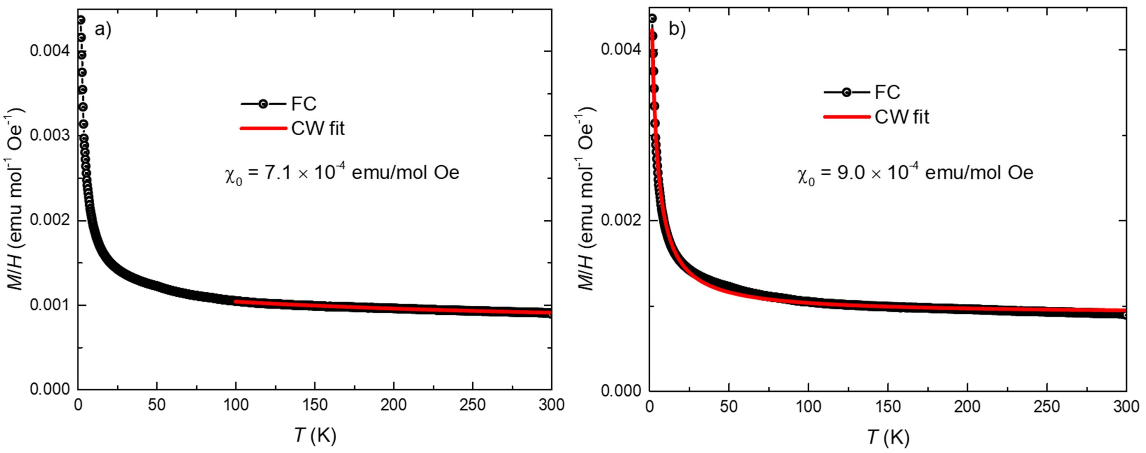

Figure S2 The Curie-Weiss fittings on the DC magnetization measurements. The Curie-Weiss fittings on the DC magnetization measurements in the different temperature ranges, **a**), 100 K - 300 K. **b**) 1.8 K - 300 K. The black dots denote the measured data, and the red line gives the calculated fitting.

The Curie-Weiss model, characterized by the equation $\chi = C/(T - \theta) + \chi_0$, has been employed to fit the magnetization-temperature ($M(T)$) data under an applied magnetic field of 1 *k*Oe for a polycrystalline $Pb_5(PO_4)_3OH_\delta$ powder, as shown in Fig. S2. Here, χ represents the magnetic susceptibility, C is the Curie constant, T is the temperature, θ is the Weiss temperature, and χ_0 is a temperature-independent susceptibility term. The fitting yields an effective magnetic moment (μ_{eff}) of approximately 0.907 μ_B/f.u. for the 50 -300 K temperature range and 0.987 μ_B/f.u. for the 1.8 -300 K temperature range.

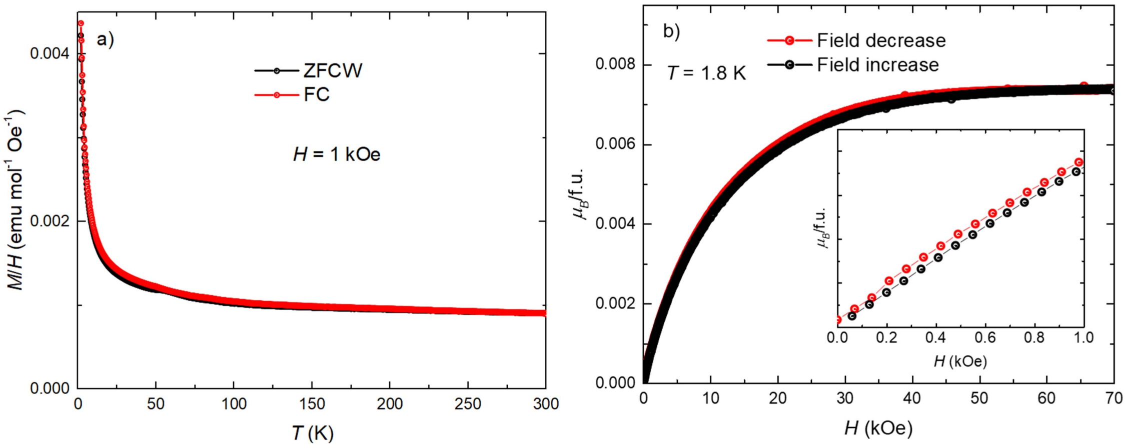

Figure S3 Temperature- and field-dependent of $Pb_5(PO_4)_3OH_\delta$ magnetization data. **a**, Zero-field-cooled-warming (ZFCW) and field-cooled (FC) magnetization as a function of temperature for the $Pb_5(PO_4)_3OH_\delta$ with a field of 1 kOe applied parallel. **b**, Magnetization as a function of the field is shown at a temperature of 1.8 K. The right inset in **b** shows a low field range of $M(H)$.

Table S1 ^{1}H ssNMR parameters used for data acquisition at 18.8 T with a rotor-synchronized Hahn echo.

**Material**	ν_{rot} (kHz)	**Time Domain** (points)	**Spec. Width** (kHz)	**Dwell Time** (μs)	**Acq. Time** (ms)	**Recycle Delay** (s)	**π/2** (μs)	**Echo length** (μs)	**Scans**
$PbPO_4$	8	2000	100	5	10	2	2.5	500	16
Alanine	10	2000	100	5	10	2	2.5	400	16
